# The effect of different combinations of vascular, dependency and cognitive endpoints on the sample size required to detect a treatment effect in trials of treatments to improve outcome after lacunar and non-lacunar ischaemic stroke

**DOI:** 10.1177/2396987317728854

**Published:** 2017-09-05

**Authors:** Stephen DJ Makin, Fergus N Doubal, Terence J Quinn, Philip MW Bath, Martin S Dennis, Joanna M Wardlaw

**Affiliations:** 1 Centre for Clinical Brain Sciences, Chancellors Building, Edinburgh, UK; 2 Academic Section of Geriatric Medicine, Institute of Cardiovascular and Medical Sciences, 3526 University of Glasgow, Glasgow, UK; 3 UK Dementia Research Institute, 3124 University of Edinburgh, Edinburgh Medical School, Edinburgh, UK; 4 Stroke Trials Unit, Division of Clinical Neuroscience, 6123 University of Nottingham, Nottingham, UK

**Keywords:** Stroke, randomised trial, sample size, power calculation, lacunar, cognition, dependency, outcome

## Abstract

**Background:**

Endpoints that are commonly used in trials of moderate/severe stroke may be less frequent in patients with minor, non-disabling stroke thus inflating sample sizes. We tested whether trial efficiency might be improved with composite endpoints.

**Methods:**

We prospectively recruited patients with lacunar and minor non-lacunar ischaemic stroke (NIHSS ≤ 7) and assessed recurrent vascular events (stroke, transient ischaemic attack (TIA), ischemic heart disease (IHD)), modified Rankin Score (mRS) and cognitive testing with the Addenbrooke’s Cognitive Examination (ACE-R) one year post-stroke. For a potential secondary prevention randomised controlled trial (RCT), we estimated sample sizes using individual or combined outcomes, at power 80% (and 90%), alpha 5%, required to detect a relative 10% risk reduction.

**Results:**

Amongst 264 patients (118 lacunar, 146 non-lacunar), at one year, 30/264 (11%) patients had a recurrent vascular event, 5 (2%) had died, 3 (1%) had clinically-diagnosed dementia, 53/264 (20%) had mRS ≥ 3 and 29/158 (19%) had ACE-R ≤ 82 (57 could not attend for cognitive testing). For a potential trial, at 80% power, using mRS ≥ 3 alone would require *n* > 5000 participants, recurrent vascular events alone *n* = 9908 participants, and a composite of any recurrent vascular event, ACE-R ≤ 82, dementia or mRS ≥ 2 (present in 56% of patients) *n* = 2224 patients. However, including cognition increased missing data. Results were similar for lacunar and non-lacunar minor ischaemic stroke.

**Conclusions:**

Composite outcomes including vascular events, dependency, and cognition reduce sample size and increase efficiency, feasibility, and relevance to patients of RCTs in minor ischaemic stroke. Efficiency might be improved further with more practical cognitive test strategies.

## Introduction

The endpoints commonly used in trials of treatments for moderate and severe stroke,^1^ such as death or dependency (often measured on the modified Rankin Scale (mRS)),^2^ may occur less frequently in patients with minor stroke and, therefore, inflate the sample size required in a randomised controlled trial (RCT). Such trials might include testing treatments for lacunar stroke, an important but neglected subtype of ischaemic stroke for which currently there is no specific treatment, but where trials are planned.^3^

Although death or dependence^2^ is important, other individual outcomes may also be of concern to patients with minor stroke, such as cognitive decline. Combining outcome measures into a composite outcome has the potential to increase trial efficiency by increasing the proportion of patients with the endpoint, improving power at smaller sample sizes and reducing costs and trial duration. Combined outcomes may also provide an overall outcome which captures several factors of relevance to patients.

We used data from a longitudinal observational study of patients with a lacunar or minor non-lacunar ischaemic stroke to test the effect of several possible single and composite outcomes, assessed at one year after index stroke, on sample size estimates for RCTs.

## Methods

We recruited consecutive inpatients and outpatients who presented to our Regional Stroke Service with a lacunar or minor non-lacunar ischaemic stroke. ‘Minor’ stroke was defined as NIHSS ≤ 7 and expected to be non-disabling at the point of assessment, i.e. recovery to no disability in basic activities of daily living (ADLs)^4^ like washing, dressing walking, bathing, but which might cause some reduction in instrumental ADLs.^5^ We recorded patient characteristics and medical history including vascular risk factors at recruitment, as reported previously.^5^

The study was approved by Lothian Research Ethics committee (REC 09/81,101/54) and NHS Lothian R + D Office (2009/W/NEU/14), and all patients gave written informed consent.

We introduced cognitive testing with the Addenbrooke’s cognitive examination-revised version (ACE-R)^6^ at one month and one year after stroke; cognitive testing did not start until after the first 56 patients had been recruited due to delays in obtaining ethics approval. We considered a score of ≤82 to indicate cognitive impairment as it was the cut-off recommended in a validation paper as having a high specificity for dementia.^6^ The ACE-R is a multi-domain cognitive screening tool, similar to the Montreal Cognitive Assessment (MoCA) in many respects including its sensitivity and specificity for dementia and multi-domain cognitive impairment in the post-stroke setting.^7^

We followed-up all patients face-to-face at one year post-stroke to identify any history suggestive of recurrent stroke, TIA, ischaemic heart disease (IHD) whether new episode of angina, or myocardial infarction during follow-up, performed physical examination including NIHSS and blood pressure, and measured the modified Rankin scale (mRS) using the structured method.^8^ If patients were unable to attend we performed telephone assessment, and if that was impossible we obtained relevant information from carers or the family doctor.

### Statistical analysis

We used R statistical software (R Foundation for Statistical Computing, Vienna, Austria, http://www.R-project.org/) to run Fisher’s exact test (dichotomous variables) and the Mann–Whitney U test (continuous non-parametric variables) in univariate analyses to compare the characteristics in patients with lacunar and non-lacunar stroke.

We calculated the sample size required to detect a 10% relative risk reduction in the outcome of interest, at 80% and 90% power, these effect sizes being similar to that of several commonly used medical interventions, e.g. antiplatelets for secondary stroke prevention.^9^ For example, if an outcome occurred in 40% of participants, we calculated the sample size required to detect a reduction of 4%, from 40% to 36%. If an outcome occurred in 5% of participants, we calculated the sample required to detect a reduction of 0.5%, from 5% to 4.5%.

We performed all sample size calculations for powers of 80% and 90%, as these are two conventional values.^10^ We calculated the sample size for lacunar and non-lacunar stroke separately, and then for all stroke combined.

We first tested single outcomes, e.g. ‘recurrent stroke’ or, ‘ACE-R ≤ 82,’ and then tested combinations of vascular events, e.g. ‘recurrent stroke or TIA.’ We then incorporated dependence into the outcomes (testing both mRS ≥ 2 and mRS ≥ 3), and finally we incorporated cognition e.g. ‘Stroke, TIA, IHD, ACE-R ≤ 82, dementia, death or mRS ≥ 2.’ We then tested outcomes that included cognition, dependency and death but not recurrent vascular events, e.g. ‘ACE-R ≤ 82, dementia, death or mRS ≥ 3’ to allow for RCTs of differing objectives and agents. We included dementia as well as ACE-R ≤ 82 since dementia, a clinical diagnosis, might be available in a patient who was not able to undergo trial-based cognitive test like the ACE-R. A composite endpoint is a binary outcome measure: it is considered to have occurred if a patient had one or more of the component endpoints: for example if a patient had either a recurrent stroke, or a TIA, or both a stroke and TIA we would consider that they had experienced the endpoint ‘Stroke or TIA.’

## Results

**Figure 1.**
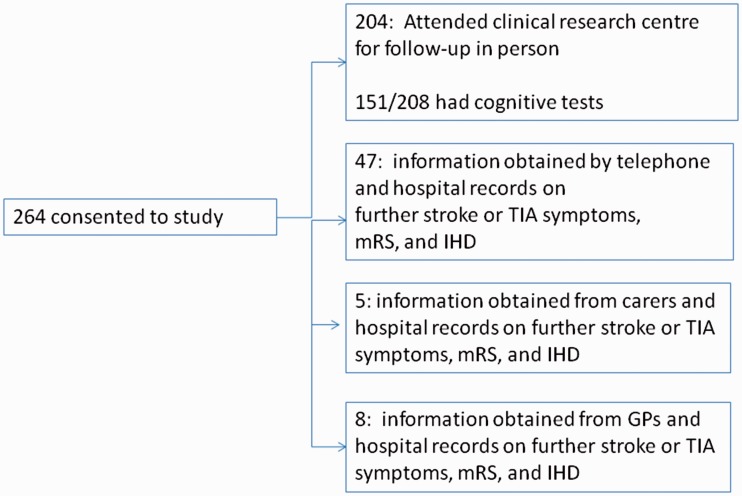
Recruitment and follow-up.

We screened 471 patients with a potential diagnosis of minor ischaemic stroke and recruited 264 (details, Figure 1).^5^ About 208 patients had cognitive testing at baseline since cognitive testing was introduced after the first 56 patients were recruited.

### Patient characteristics and rates of individual outcomes

At baseline (Table 1) the median age was 67 (range 36–98), 110/264 (42%) were female, the median NIHSS was 2 (interquartile range, IQR 1–3) and 118/264 (45%) patients had a lacunar stroke. The median mRS at the time of initial cognitive assessment was 1 (IQR 0-2).

**Table 1.** Characteristics of patients at baseline and at one year.

	Lacunar *n* = 118	Non-lacunar *n* = 146	*p*	All ischaemic stroke
Median age years (IQR)	64(55–65)	69(61–77)	**0.0063**	67(59–67)
Female gender (%)	51(43%)	59(41%)	0.71	110(42%)
Previous TIA	11(9%)	17(12%)	0.68	28(11%)
Previous stroke	16(14%)	16(11%)	0.57	32(12%)
Ischaemic heart disease (IHD)	19(16%)	34(23%)	0.17	53(20%)
Diagnosis of peripheral vascular disease (PVD)	3(3%)	12(8%)	**0.032**	15(6%)
Diabetes	12(10%)	18(12%)	0.70	30(11%)
Hypertension	81(69%)	106(72%)	0.50	187(71%)
Atrial fibrillation (AF)	7(6%)	18(12%)	0.09	25(9%)
Diagnosis of hyperlipidaemia prior to index stroke, or at presentation	73(62%)	88(60%)	0.80	161(62%)
Current smoker	46(39%)	44(30%)	0.15	90(34%)
Median NIHSS (IQR)	2(2–4)	2(1–3)	**0.0092**	2(1–3)
Median systolic BP (IQR)	147(130–158.5)	138(125.5–159)	0.16	142.5(130–159)
Median mRS (IQR) at baseline assessment	1(1–2)	1(1–2)	0.67	1(1–2)
Characteristics at 1 year				
Diagnosis of dementia	1(1%)	2(1%)	1.00	3(1%)
IHD in the year following the stroke (e.g. ongoing angina, or new myocardial infarction)	14(12%)	22(15%)	0.48	36(14%)
NIHSS at 1 year (IQR)	0(0–1)	0(0–0)	0.72	0(0–0.25)
NIHSS at 1 year ≥1	23(26%)	27(24%)	0.75	50(19%)
mRS (IQR)	1(0–2)	1(1–2)	0.12	1(1–2)
mRS = 0 (No symptoms)	30(21%)	32(27%)	0.24	62(23%)
mRS ≥ 1 (Some symptoms)	86(73%)	116(79%)	0.24	202(77%)
mRS ≥ 2	47(40%)	71(49%)	0.17	118(45%)
mRS ≥ 3	20(17%)	33(23%)	0.28	53(20%)
New TIA	3(3%)	4(3%)	1	7(3%)
New stroke	10(8%)	15(10%)	0.68	25(9%)
Either new stroke or TIA	12(10%)	18(12%)	0.70	30(11%)
ACE-R at 1 year median (Interquartile range) in *n* = 151 tested at 1 year	92(71–96)	90(59–94)	0.54	91(59–95)
ACE-R ≤ 82 in *n* = 151 tested at 1 year	14(22%)	15(17%)	0.53	29(19%)

Bold = *p* values that indicate significant differences at *p* < 0.01 between lacunar and cortical stroke subgroups.

At one year, we followed up all 264 patients to ascertain if they were alive or dead, had had a recurrent vascular event, and their functional status; patients seen in person underwent repeat cognitive assessment. We assessed 204 in person, 47 by telephone, five via carers or relatives, and eight via their GP (Figure 1).

At one year, 30 (11%) patients had had a recurrent stroke or TIA, 5 (2%) had died, and 3 (1%) had been diagnosed clinically with dementia. Many patients 118/264 (45%) still had some symptoms of stroke and 53/264 (20%) required assistance from family or carers with activities of daily living at least once per week. More patients with non-lacunar stroke had new diagnosis of peripheral vascular disease during follow-up(10/146 patients with non-lacunar stroke v 0/118 patients with lacunar stroke), there were no other statistically significant differences in outcomes between patients with lacunar or non-lacunar stroke.

Of the 208 patients recruited after cognitive testing was introduced, 151/208 were tested at one year of whom 29/151 (19%) had an ACE-R ≤ 82. Of the 57/208 patients not having one year cognitive testing, 3 had died, 32 declined further testing, 19 were too unwell, and 3 had visual or language disabilities precluding testing.

### Sample size estimations

The effect of several single and composite endpoints on sample size, at 80% and 90% power, is shown in Table 2. For example, 10% of patients had a recurrent stroke (26/264), so to detect a 10% relative reduction in recurrent stroke at one year (from 26/264 to 23/264) would require 29,818 patients at 80% power. A larger proportion of patients, 29/151, 19% of those cognitively tested, had an ACE-R ≤ 82 at one year, which would require a sample size of 12,570 to detect a 10% reduction. However, 118/264 (45%) of patients had a mRS ≥ 2, therefore a sample size of 3864 would be required to detect a 10% reduction in mRS ≥ 2. The sample size estimations were similar for patients with lacunar and non-lacunar stroke since the proportion of most outcomes (Table 1) was similar in these two stroke subtypes (supplementary information).

**Table 2.** Estimated sample size required to detect a 10% reduction in event rate for various combined outcomes at 80% power. Full details of individual and different combinations of outcomes at 80% and 90% power for lacunar and non-lacunar stroke at two mRS cut points are given in Supplementary Table 1.

	Sample size required	Sample size if combined with mRS ≥ 3	Sample size if combined with mRS ≥ 2 AND ACE ≤ 82^a^
Recurrent stroke or TIA	23,600	7958	3090
Recurrent stroke or TIA or IHD	9908	4398	2224
Recurrent stroke, TIA, IHD, death Similar to the major adverse cardiovascular events (MACE) endpoint used in cardiovascular trials.	9144	4398	2224

a Includes clinical diagnosis of dementia.

For composite outcomes at one year, such as ‘recurrent stroke or TIA, new IHD, or death,’ 25% would have the outcome, requiring a sample size of 9144 to be followed up to one year at 80% power (Table 2; 12,240 patients at 90% power, Supplementary Table 1). At 80% power, adding mRS ≥ 3 to this composite reduced the sample size to approximately half (4398) and replacing mRS ≥ 3 with mRS ≥ 2 reduced the sample size to approximately a third (3126) of 9144. Then, adding ACE-R ≤ 82 to the composite outcome of ‘stroke, TIA, new IHD, dementia diagnosis, death or an mRS ≥ 2,’ which occurred in 56% of patients without any double counting, reduced the sample size to 2224 patients.

**Figure 2.**
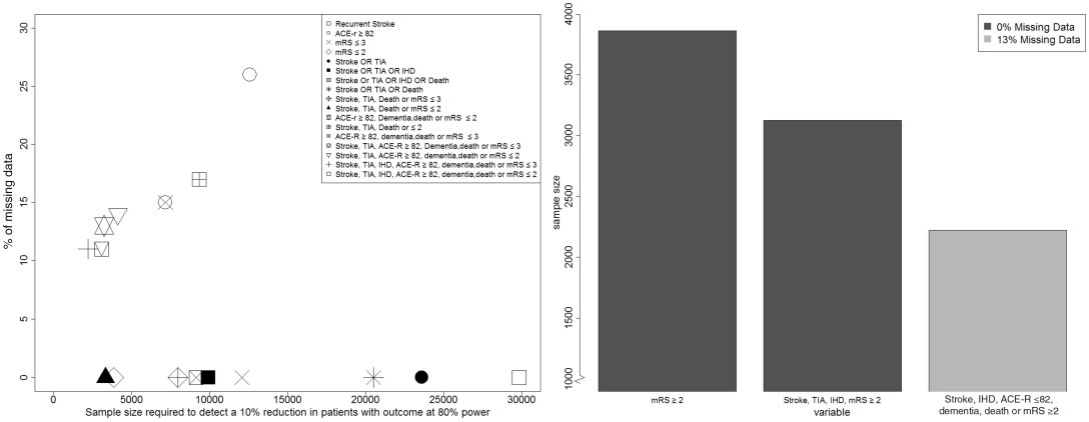
The effect of adding variables to a combined outcome on overall sample size required to detect a 10% reduction at 80% power.

Although including ACE-R in the composite outcome reduced the sample size, it introduced missing data: 57/208 (27%) patients recruited with baseline cognitive testing could not have cognitive testing at one year mainly for medical reasons. There were fewer missing outcomes when considered as part of a composite endpoint: although 27% of patients had a missing outcome for ‘ACE-R ≤ 82,’ only 11% had missing data for ‘stroke, TIA, IHD, ACE-R ≤ 82, dementia, death or mRS ≥ 2.’ Figure 2 illustrates that while composite endpoints that include ACE-R do help to reduce sample size, up to 30% of the cognitive data may be missing, whereas composites that do not include cognition have very little missing data but need larger sample sizes.

One way to compensate for missing data is to increase the sample size recruited (Supplementary Table 2). If retention progresses in a similar way to MSS-2 then to detect a 10% reduction in ‘stoke, TIA, dementia, death, mRS < /=2 or ACE-R > /=82,’ 2499 patients would need to be recruited in order for 2106 to be followed up at one year.

Another method is to use the ‘last observation carried forward’ (LOCF) method. About 157 patients had cognitive testing at 1–3 months post stroke of which 36 had ACE-R ≤ 82. By one year, 19/36 had ACE-R ≥ 82, 8 had an ACE-R < 82 and 9 were not tested; in contrast, of the 121 patients who had ACE-R < 82 at 1–3 months 4 had an ACE-R ≤ 82, at one year and 13 were not tested. If the LOCF method is used for the missing cognitive data, then 126/208 patients have the outcome measure of stroke, TIA, dementia, death, mRS>/=2 or ACE-R ≤ 82 at one year, with only 9% of patients lost to follow-up at one year. This suggests that one year data on 1748 patients would need to be recorded to detect a 10% reduction in outcome rate, at 80% power and 1880 would need to be recruited in order to follow-up 1748 patients at one year.

## Discussion

We demonstrate that in patients with minor ischaemic stroke, in whom some important individual outcome events are infrequent, that a composite outcome such as ‘recurrent stroke, TIA, new IHD, ACE-R ≤ 82, new diagnosis of dementia or an mRS ≥ 2’ produced an outcome event rate of 56% and hence could substantially reduce sample sizes required to detect modest but worthwhile treatment effects while retaining conventional power. The net effect would be smaller, less expensive and more rapidly completed RCTs in subtypes of stroke that are less common therefore provide a smaller pool from which to recruit, and have been less studied to date.

Adding mRS ≥ 3 halved the sample size from that based on recurrent stroke/TIA/IHD; using mRS ≥ 2 reduced it to a third; and adding in ACE-R ≤ 82 reduced it to an eighth of the starting sample. There was little difference between samples calculated for lacunar and non-lacunar stroke because the proportions of outcomes in these minor stroke patients were similar. These calculations were based on patients with similar characteristics to patients currently being recruited to a RCT testing interventions to prevent progression of small vessel disease in patients with lacunar stroke (LACI-1, NCT02481323) and to those who were recruited in the Secondary Prevention of Small Subcortical Stroke (SPS3) trial in lacunar stroke.^11^ However, the data came from a single population at a single centre that may limit generalisation to other settings. A similar exercise should be undertaken in other populations since outcome rates may differ.

Despite these benefits, composite outcomes may also have drawbacks that should be considered carefully. Interpretation may be more difficult since analyses based on composite outcomes generally emphasise the first event so a minor initial outcome can mask a subsequent major one.^12^ Additionally, it is theoretically possible for a treatment to have a positive effect on one outcome, and a negative effect on another, so a neutral trial result may mask a clinically significant outcome. This can be partly mitigated by careful presentation of results so that both individual and composite outcomes are easily visible to the reader. It is also important to choose components that are both relevant to patients and are biologically plausible (e.g. cognitive rehabilitation may reduce dependence but not recurrent stroke).

The combined outcome approach suggested here assumes that all outcomes are equally significant and of equal weight. Further data are needed to suggest, for instance, whether patients consider myocardial infarction to be as severe as a diagnosis of dementia. Other approaches, such as weighting the different outcome measures based on their relevance to patients, could be tested in future work. Example approaches include ordinalising recurrent events (as used in the TARDIS trial^13,14^), using global tests that integrate individual outcomes statistically (e.g. using the Wald or Wei–Lachin tests^15,16^) or using Pocock’s Win Ratio.^17^

Including cognitive testing in the outcome of any stroke RCT introduces attrition bias, as some patients are unable to have cognitive testing at follow-up for various reasons, even when a relatively simple screening tool is used, as here (Figure 2). This leads to missing data and significant underestimation of post-stroke cognitive impairment.^18^ Missing data is a challenge for any clinical trial; whilst increasing the number of patients recruited and using the LOCF method can ensure that the necessary number reach one year follow-up, both methods may increase bias. Patients that do return for follow-up are unrepresentative of those that do not return, and the use of LOCF assumes that cognition is static from one to three months to one year post-stroke. How missing data is handled can have a substantial impact on results, the method used should be explicit in the protocol and statistical analysis plan, and not decided post-hoc.^19^ The SPS3 trial^20^ managed to achieve more complete follow-up – with only 11% of patients having missing cognitive tests at five-year follow-up. However, they only recruited patients who were able to have baseline cognitive tests in the sub-acute phase, and our patients were recruited a median of four days post-stroke.

Telephone cognitive assessment could reduce attrition bias, although is only applicable to some aspects of cognition. Several phone tests, at various stages of validation, are available.^21^ However, these do not allow for multi-domain screening of visuospatial and certain aspects of executive function.^22^ Even with a telephone assessment of cognition, there would still have been more missing outcomes than for other endpoints, underlining the problem of assessing cognition after stroke. Whilst the sample size could be increased to account for the expected proportion of patients with missing outcomes, such a sample may still be biased towards the healthier patients. Further research is needed to estimate required sample size if telephone cognitive testing is used. Self-reported outcome measures are available for several stroke-related outcomes^23^ and more are needed.^24,25^

For a common condition such as a stroke, a relatively small reduction in adverse outcomes would benefit a large number of people and therefore small effect sizes are worth trying to detect reliably with the smallest sample and shortest duration of follow-up possible, to reduce trial costs and minimise participant and researcher trial fatigue. Composite outcomes have the potential to do this and so accelerate trials of potential treatments; however, they can be more challenging to interpret, and care needs to be given when considering how to handle missing data.

## Supplementary Material

Supplementary material
