# Supplementary material for: The effect of different combinations of vascular, dependency and cognitive endpoints on the sample size required to detect a treatment effect in trials of treatments to improve outcome after lacunar and non-lacunar ischaemic stroke
Source: Eur Stroke J. 2017 Sep 5;3(1):66–73. doi: 10.1177/2396987317728854 (PMC5992736; doi:10.1177/2396987317728854)
Supplement: Supplementary material [file ESO728854_supplementary_tables.pdf]

Supplementary information

Supplementary Table 1 Effect on sample size of individual and composite outcomes in lacunar and non-lacunar ischaemic stroke or both subtypes combined at 80% and 90% power.

	**Lacunar n=118**			**Non-lacunar n=146**			**All Ischaemic Stroke**			**Missing Data (%)**
	N (%)	90% Power	80% power	N (%)	90% Power	80% power	n (%)	90% Power	80% power	
Stroke	10 (8%)	43038	32938	14 (10%)	37764	28208	24 (9%)	39918	29818	0
TIA	2 (2%)	260140	194320	4 (3%)	146310	109292	6 (2%)	182734	136498	0
IHD in the year following the stroke (e.g. ongoing angina, or new myocardial infarction)	14 (12%)	30184	22548	22 (15%)	22608	16888	36 (14%)	25918	19360	4
Dementia	1 (1%)	414454	309590	2 (1%)	275218	205582	3 (1%)	369492	276004	1
Death	2 (2%)	260140	194320	3 (2%)	200950	150106	5 (2%)	205284	153344	0
mRS ≥3	20 (17%)	19642	14672	33 (23%)	13868	10360	53 (20%)	16166	12076	0
mRS ≥2	47 (40%)	6234	4656	71 (49%)	4414	3298	118 (45%)	5172	3864	0
ACE-R ≤ 82	14(22%)	14198	10606	15 (17%)	19504	14570	29 (19%)	16826	12570	57 (27%)
Stroke or TIA	12 (10%)	35256	26336	18 (12%)	28732	21462	30 (11%)	31594	23600	0
Stroke, TIA or IHD	25 (21%)	15030	11228	37 (25%)	11996	8962	62 (23%)	13264	9908	0
Stroke, TIA, IHD or Death	27 (23%)	13628	10180	39 (27%)	11158	8336	66 (25%)	12240	9144	0
Stroke, TIA or death	14 (12%)	30184	22548	20 (14%)	25350	18936	34 (13%)	27458	20510	0

Stroke, TIA, death or mRS≥ 3	29 (25%)	12424	9282	44 (30%)	9410	7030	73 (28%)	10654	7958	0
Stroke, TIA, death or mRS ≥ 2	51 (43%)	5442	4066	77 (53%)	3774	2820	128 (48%)	4466	3336	0
Stroke, TIA, IHD, Death, or mRS ≥3	39(33%)	10070	7522	57(39%)	6116	4568	96(36%)	5886	4398	0
Stroke, TIA, IHD, Death, or mRS ≥2	58(49%)	4174	3118	88(60%)	2852	2132	146(55%)	4184	3126	0
Stroke, TIA, dementia, death mRS ≥3, or ACE-R≤82	21 (33%)	8194	6122	24 (27%)	10900	8142	45 (30%)	9598	7170	31 (15%)
Stroke, TIA, ACE -R≤82, dementia, death mRS ≥2, or ACE-R≤82,	33 (52%)	3818	2852	43 (49%)	4382	3274	76 (50%)	4136	3090	24 (11%)
Stroke, TIA, IHD, dementia, death, mRS ≥3 or ACE-R≤82	27 (43%)	5504	4112	34 (39%)	6512	4864	61 (40%)	6082	4128	29 (14%)
Stroke, TIA, IHD, dementia, death, mRS	38 (60%)	2820	2106	51 (58%)	3094	2312	89 (59%)	2976	2224	23 (11%)

≥2 or ACE-R≤82										
**End points which DO NOT include recurrent vascular events**										
ACE-R≤82 or dementia	14 (22%)	14198	10606	15 (17%)	19504	14570	29 (19%)	16826	12570	55 (26%)
ACE–R≤82, dementia, death or mRS ≥3	19 (30%)	9414	7032	18 (20%)	15806	11806	37 (25%)	12498	9336	35 (17%)
ACE-R≤82, dementia, death or mRS ≥2	33 (52%)	3818	2852	41 (47%)	4766	3560	74 (49%)	4348	3248	28 (13%)

	Missing Data (%)	No that would need to be recruited to record outcome data on the no of patients calculated in Table S1		No of patients with outcome if the last observation carried forward is used	No of patients whose one year follow-up data would need to be recorded in order to detect a 10% reduction in outcome if LOCF was used		No of patients with missing data if LOCF was used (%)	No that would need to be recruited to for the no of patients followed up.	
				n (%)	90% Power	80% Power		90% Power	80% power
Stroke	0	39918	29818	24 (9%)	39918	29818		39918	29818
TIA	0	182734	136498	6 (2%)	182734	136498		182734	136498
IHD in the year following the stroke (e.g. ongoing angina, or new myocardial infarction)	4	26998	20167	36 (14%)	25918	19360		25918	19360
Dementia	1	373224	278792	3 (1%)	369492	276004		369492	276004
Death	0	205284	153344	5 (2%)	205284	153344		205284	153344
mRS ≥3	0	16166	12076	53 (20%)	16166	12076		16166	12076
mRS ≥2	0	5172	3864	118 (45%)	5172	3864		5172	3864
ACE-R ≤ 82	57 (27%)	23049	17219	38(22%)	14350	10720	35(17%)	17289	12916
Stroke or TIA	0	31594	23600	30 (11%)	31594	23600		31594	23600
Stroke, TIA or IHD	0	13264	9908	62 (23%)	13264	9908		13264	9908
Stroke, TIA, IHD or Death	0	12240	9144	66 (25%)	12240	9144		12240	9144
Stroke, TIA or death	0	27458	20510	34 (13%)	27458	20510		27458	20510
Stroke, TIA, death or mRS≥ 3	0	10654	7958	73 (28%)	10654	7958		10654	7958

	Missing Data (%)	No that would need to be recruited to record outcome data on the no of patients calculated in Table S1		No of patients with outcome if the last observation carried forward is used	No of patients whose one year follow-up data would need to be recorded in order to detect a 10% reduction in outcome if LOCF was used		No of patients with missing data if LOCF was used (%)	No that would need to be recruited to for the no of patients followed up.	
				n (%)	90% Power	80% Power		90% Power	80% power
Stroke, TIA, death or mRS ≥ 2	0	4466	3336	128 (48%)	4466	3336		4466	3336
Stroke, TIA, IHD, Death, or mRS ≥3	0	5886	4398	96(36%)	5886	4398		5886	4398
Stroke, TIA, IHD, Death, or mRS ≥2	0	4184	3126	146(55%)	4184	3126		4184	3126
Stroke, TIA, dementia, death mRS ≥3, or ACE-R≤82	31 (15%)	11292	8435	74(40%)	6182	4618	21(11%)	6946	5189
Stroke, TIA, dementia, death mRS ≥2, or ACE-R≤82,	24 (11%)	4647	3472	112(68%)	2070	1546	16(8%)	2250	1680
Stroke, TIA, IHD, dementia, death, mRS ≥3 or ACE-R≤82	29 (14%)	7072	4800	92(48%)	4518	3374	19(9%)	4965	3708

	Missing Data (%)	No that would need to be recruited to record outcome data on the no of patients calculated in Table S1		No of patients with outcome if the last observation carried forward is used	No of patients whose one year follow-up data would need to be recorded in order to detect a 10% reduction in outcome if LOCF was used		No of patients with missing data if LOCF was used (%)	No that would need to be recruited to for the no of patients followed up.	
				n (%)	90% Power	80% Power		90% Power	80% power
Stroke, TIA, IHD, dementia, death, mRS ≥2 or ACE-R≤82	23 (11%)	3344	2499	126(65%)	2340	1748	15(7%)	2516	1880
	**End points which DO NOT include recurrent vascular events**								
ACE-R≤82 or dementia	55(26%)	22738	16986	31(18%)	18386	13734	34(16%)	21888	16350
ACE–R≤82, dementia, death or mRS ≥3	35 (17%)	15058	11248	63(34%)	7944	5934	24(11%)	8926	6667
ACE-R≤82, dementia, death or mRS ≥2	28(13%)	4998	3733	107(57%)	3204	2394	19(9%)	3521	2631
